# Parâmetros Ecocardiográficos Simples são Fortes Preditores de Risco Cardiovascular em Indivíduos Assintomáticos: Coorte Elsa-Brasil

**DOI:** 10.36660/abc.20210101

**Published:** 2022-05-04

**Authors:** Luciana Pereira Fernandes, Maria da Conceição Chagas de Almeida, Sheila Alvim de Matos, Ana Clara Paixão Campos, Edmundo José Nassri Câmara, Murilo Foppa, Antônio Luiz Pinho Ribeiro, Sandhi Maria Barreto, Roque Aras

**Affiliations:** 1 Universidade Federal da Bahia Salvador BA Brasil Universidade Federal da Bahia, Salvador, BA – Brasil; 2 Fundação Oswaldo Cruz Instituto Gonçalo Moniz Salvador BA Brasil Fundação Oswaldo Cruz - Instituto Gonçalo Moniz, Salvador, BA – Brasil; 3 Universidade Federal do Rio Grande do Sul Porto Alegre RS Brasil Universidade Federal do Rio Grande do Sul, Porto Alegre, RS – Brasil; 4 Universidade Federal de Minas Gerais Belo Horizonte MG Brasil Universidade Federal de Minas Gerais, Belo Horizonte, MG – Brasil

**Keywords:** Doenças Cardiovasculares, Fatores de Risco, Disfunção Diastólica Ventricular Esquerda, Volume do Átrio Esquerdo, Diagnóstico por Imagem, Ecocardiografia/métodos, Aterosclerose, Sedentarismo

## Abstract

**Fundamento:**

vários estudos avaliam alterações ecocardiográficas como preditores de risco cardiovascular; entretanto, nenhum associa risco cardiovascular global com alterações ecocardiográficas em brasileiros.

**Objetivo:**

Este estudo avalia a associação entre risco cardiovascular global (ASCVD) e achados ecocardiográficos como hipertrofia ventricular esquerda (HVE), disfunção diastólica (DDVE) e aumento do volume do átrio esquerdo (AE).

**Métodos:**

A população foi composta por participantes do ELSA-Brasil que realizaram ecocardiografia entre 2008 e 2010 (n = 2.973). Eram assintomáticos e não tinham história de doença cardiovascular (DCV). O escore ASCVD foi calculado em dois períodos: 2008-2010 e 2012-2014. Razões de prevalência (RP) foram estimadas com intervalos de confiança (IC) de 95%.

**Resultados:**

Evidenciou-se associação entre alterações ecocardiográficas e alto risco cardiovascular global (escore ASCVD ≥ 7,5) nos dois períodos do estudo, separadamente. O risco global combinado (baixo risco no primeiro período e alto risco no segundo período) teve associação significativa apenas com DDVE (RP = 3,68; IC 95%: 2,63-5,15) e HVE (RP = 2,20; IC 95%: 1,62–3,00).

**Conclusão:**

Alterações ecocardiográficas (DDVE, HVE e aumento do volume do AE) são preditores independentes de risco cardiovascular em adultos brasileiros sem DCV prévias.

## Introdução

As doenças cardiovasculares (DCV) são um problema de saúde global e uma prioridade de pesquisa em muitos países.^1^ No Brasil, o Estudo Longitudinal de Saúde do Adulto (ELSA-Brasil) visa investigar a incidência de doenças crônicas não transmissíveis, especialmente DCV, e seus fatores de risco na população adulta.^2^ Nesse contexto, merece investigação a identificação de preditores de risco cardiovascular (CV).

Atualmente, o escore de risco CV mais utilizado universalmente é o de Doença Cardiovascular Aterosclerótica (ASCVD), cujos parâmetros foram definidos por estudos realizados nos Estados Unidos da América.^3^ Outros estudos avaliaram a capacidade preditiva das alterações ecocardiográficas.^4 , 5^ No entanto, nenhum estudo investigou a associação do escore ASCVD com alterações ecocardiográficas na população brasileira.

Assim, considerando o escore ASCVD como desfecho CV intermediário, o presente estudo avaliou associação de alterações ecocardiográficas com o escore ASCVD em indivíduos assintomáticos sem DCV prévia em dois períodos do ELSA-Brasil: basal (período 1) e 4 anos depois (período 2).

## Métodos

### População

A população foi composta por participantes do ELSA-Brasil que realizaram ecocardiograma entre 2008 e 2010. Esses indivíduos faziam parte de duas amostras, uma aleatória, composta por 10% da coorte (n = 15.105) e outra, composta por indivíduos maiores de 60 anos não incluídos na amostra aleatória. Foram excluídos aqueles que referiram DCV no início do estudo (disfunção ventricular esquerda, infarto do miocárdio, acidente vascular cerebral, fibrilação ou flutter atrial e doença valvar moderada ou grave).

Para calcular o escore ASCVD, foram extraídos dados produzidos em 2008–2010 e 2012–2014 pelo ELSA-Brasil, conforme descrito alhures.^6^ A ecocardiografia foi realizada apenas no primeiro período.

Sendo um estudo multicêntrico, o protocolo de pesquisa foi aprovado não apenas pelo comitê de ética de cada instituição, mas também pela Comissão Nacional de Ética em Pesquisa.

### Ecocardiografia

A ecocardiografia foi realizada por profissionais credenciados em aparelho do mesmo modelo (Aplio XG; Toshiba Corporation, Tóquio, Japão) nos seis centros do ELSA-Brasil, seguindo técnica padronizada. Imagens foram selecionadas e enviadas em formato DICOM (Digital Imaging Communications in Medicine) para uma central de leitura onde se realizaram as medições.^6^

Analisaram-se três parâmetros ecocardiográficos: hipertrofia ventricular esquerda (HVE), disfunção diastólica do ventrículo esquerdo (DDVE) e aumento do volume do átrio esquerdo (AE). Definiu-se HVE de acordo com dois critérios: índice de massa e espessura relativa da parede (ER). Calculou-se o índice de massa indexando a massa do VE à área de superfície corporal (ASC) ou à altura ^2 , 7^ .^7^ As medidas da massa do VE foram realizadas pela ecocardiografia bidimensional (método linear)^8^ no centro de leitura^9^ e a massa (em gramas) foi calculada pela fórmula 0,80 (1,04 [septo interventricular + dimensão interna do VE + parede posterior]^3^- [dimensão interna do VE]) + 0,6, de acordo com Devereux et al.,^10^ Calculou-se ER pela fórmula (2 x espessura da parede posterior)/(diâmetro interno do VE ao final da diástole).^8^ Usando esses critérios, classificou-se geometria do VE em normal, remodelamento concêntrico, hipertrofia concêntrica ou hipertrofia excêntrica.^11^ O ponto de corte para a massa indexada à ASC foi de 95 g/m^2^ para mulheres e 115 g/m^2^ para homens.^8^ Com a massa indexada pela altura,^2 , 7^ o ponto de corte foi 44 g/altura ^2 , 7^ para mulheres e 48 g/altura^2 , 7^ para homens.^12^ Considerando os dois critérios,^8^ o ponto de corte da ER foi de 0,42 para ambos os sexos.

Avaliação da função diastólica do VE baseou-se nas recomendações da Sociedade Americana de Ecocardiografia de 2009.^13^ As seguintes medidas foram utilizadas para classificar a função diastólica: relação E/A (relação das velocidades E e A do influxo mitral), velocidade das ondas e’ medial e lateral (avaliada com Doppler tecidual), relação E/e’ e volume do AE indexado à ASC. Adotaram-se os seguintes pontos de corte: E/A (≤ 0,8, entre 0,8 e 2,0, e ≥ 2,0), e’ medial (< 8), e’ lateral (< 10), E/e’ (≤ 8, >8 e <13 e ≥ 13) e volume indexado do AE (> 34 ml/m^2^). Com base nesses critérios, função diastólica foi classificada em normal, disfunção grau I ou alteração do relaxamento (pressão do AE normal), disfunção grau II ou pseudonormal (sinais de pressão elevada do AE), disfunção grau III ou enchimento restritivo (pressão do AE significativamente elevada).

O volume indexado do AE foi categorizado em normal (até 34 ml /m^2^), ligeiramente aumentado (entre 35 e 41 ml/m^2^), moderadamente aumentado (entre 42 e 48 ml/m^2^) e gravemente aumentado (> 48 ml/m^2^).^8^

Para analisar conjuntamente as alterações ecocardiográficas (DDVE, HVE e aumento do volume do AE), foi criada a variável “parâmetro Ecocardio”, considerado normal quando nenhuma alteração estava presente e anormal quando pelo menos uma estava presente.

### Escore de risco CV global

O escore ASCVD, que estima o risco de um evento cardiovascular fatal ou não fatal em 10 anos (baixo < 7,5% e alto ≥ 7,5%),^3^ foi calculado com base em idade, sexo, raça (branca, afro-americana e outros), colesterol total, lipoproteína de alta densidade (HDL-colesterol), pressão arterial sistólica, tratamento para hipertensão, presença de diabetes mellitus e tabagismo. Calculou-se o risco global para cada participante nos dois períodos do estudo. Calculou-se também o risco combinado, definido como a combinação de baixo risco no primeiro período e alto risco no segundo.

### Outros fatores de risco

Além das três alterações ecocardiográficas, foram avaliados atividade física, consumo de álcool, nível sérico de triglicerídeos, índice de massa corporal (IMC) e escolaridade. Quanto à atividade física, os participantes foram categorizados em sedentários/pouco ativos (<150 min/semana de atividade moderada) ou fisicamente ativos/muito ativos (pelo menos 150 min/semana de atividade moderada).^14^ Consumo de álcool foi categorizado como consumo excessivo ou não excessivo (> 210 ou <210 g de álcool por semana para homens e > 140 ou < 140 g de álcool por semana para mulheres). Quanto aos triglicerídeos, as categorias foram < 150 ou ≥ 150 mg/dl. As categorias do IMC foram: obeso (≥ 30 kg/m^2^), sobrepeso (≥ 25 e <30 kg/m^2^) ou eutrófico (<25 kg/m^2^).^15^ Categorias de escolaridade: até o ensino médio completo e ensino superior.

### Análise estatística

Inicialmente, realizou-se análise descritiva dos perfis sociodemográfico, clínico e ecocardiográfico dos participantes. Variáveis categóricas foram apresentadas como frequências absolutas e relativas. A seguir, realizou-se análise de regressão logística bivariada para verificar associação entre as características ecocardiográficas, clínicas e sociodemográficas e o risco CV global. Razões de prevalência (RP) foram estimadas com intervalos de confiança (IC) de 95%, utilizando o comando CS do STATA versão 12. Para a análise de regressão logística multivariada, utilizou-se o pacote prLogistic do software R versão 3.5.1, estimando-se as RP por meio de modelos logísticos e os IC pelos métodos delta e bootstrap.^16^ Modificação do efeito foi avaliada para as covariáveis: educação, atividade física, consumo excessivo de álcool, triglicerídeos e IMC. Aplicou-se o teste da razão de verossimilhança no modelo de regressão logística multivariada, incorporando os termos do produto (interação) entre a associação principal e cada covariável. Um valor de p <5% foi indicativo de uma mudança de efeito.

## Resultados

### Características sociodemográficas e clínicas da população no início do estudo

Após exclusão dos indivíduos que relataram ter DCV, a amostra final do estudo foi composta por 2.973 participantes, com idade média de 60,26 ± 8,89 anos, principalmente brancos e negros (56,4% e 39,9%), e a maioria com nível superior (56,7%). Características sociodemográficas e clínicas dos participantes no início do estudo são apresentadas na tabela 1 . As características clínicas dos participantes no período 2 são apresentadas na tabela S1.

**Tabela 1:** – Características sociodemográficas e clínicas dos participantes, n= 2973, na linha de base do estudo (2008- 2010)

Características sociodemográficas e clínicas	n	%
**Sexo**		
Masculino	1358	45,7
Feminino	1615	54,3
**Idade (anos)**		
35- 44	220	7,4
45- 54	487	16,4
55- 64	1240	41,7
65- 74	1025	34,5
**Raça**		
Brancos	1658	56,4
Pretos	1174	39,9
Outros	109	3,7
**Nível educacional**		
Grau superior	1686	56,7
Até o ensino médio completo	1287	43,3
**Hipertensão**		
Sim	1440	48,5
**IMC**		
Sobrepeso	1262	42,4
Obesidade	659	22,2
**Glicemia em Jejum**		
(≥126 mg/dl)	367	12,3
**Hemoglobina glicada**		
(≥6.5)	323	10,9
**Colesterol total**		
(> 200 mg/dl **)**	1847	62,2
**HDL baixo**		
Sim	509	17,1
**Triglicérides alto**		
Sim	940	31,6
**Bebedor excessivo**		
Sim	223	7,5
**Hábito de fumar**		
Ex-fumante	1047	35,2
Fumante	296	10,0
**Atividade física**		
Sedentário	1262	42,8

*IMC: índice de massa corporal; HDL: lipoproteína de alta densidade.*

### Risco CV global (escore ASCVD)

O escore ASCVD foi avaliado nos dois períodos do estudo como um desfecho clínico intermediário. Associação do risco global com parâmetros ecocardiográficos separados e agrupados foi analisada por meio de fatores sociodemográficos (escolaridade) e clínicos (atividade física, consumo de álcool, hipertrigliceridemia e IMC). Como idade, sexo, raça/cor, colesterol total, HDL-colesterol, hipertensão, diabetes mellitus e tabagismo fazem parte da construção desse escore de risco, a associação com essas variáveis não foi avaliada.

O risco global foi <7,5% (baixo) em 1.398 participantes (47%) no primeiro período e em 1.034 participantes (38,3%) no segundo período, e ≥ 7,5% (alto) em 1.575 participantes (53%) no primeiro período e em 1.665 participantes (61,7%) no segundo período. O risco combinado esteve presente em 312 participantes (23,7%).

### Características ecocardiográficas

Em 50,8% dos participantes a função diastólica foi considerada normal e em 41,8% como anormal (destes, 31,2% eram grau I). Em 7,4% dos participantes, a função diastólica ou o grau de disfunção diastólica não puderam ser determinados.

O volume do AE estava aumentado em 15,6% de 2.438 participantes.

HVE foi classificada com base em dois tipos de indexação da massa: ASC (em 2.670 participantes) e altura^2 , 7^ (em 2.651 participantes). A proporção de participantes com HVE foi maior quando utilizada a indexação por altura (18,5% versus 10,6%), principalmente às custas da hipertrofia concêntrica (11,1% versus 6,4%).

Na análise simultânea dos três parâmetros ecocardiográficos, 65,8% dos participantes apresentaram pelo menos uma e 34,2% nenhuma das três anormalidades. ( Tabela 2 )

**Tabela 2:** – Características ecocardiográficas dos participantes no período 1, n = 2.973

Características	n	%
**Função diastólica (n=1.384)**		
Sem disfunção	703	50,8
Disfunção tipo I	432	31,2
Disfunção tipo II	147	10,6
Indeterminado	102	7,4
**Volume do AE (n=2.438)**		
Normal	2058	84,4
Levemente aumentado	281	11,5
Moderadamente aumentado	73	3,0
Gravemente aumentado	26	1,1
**Geometria do VE (massa/ASC) (n=2.670)**		
Normal	1449	54,3
Remodelamento concêntrico	940	35,2
Hipertrofia concêntrica	170	6,4
Hipertrofia excêntrica	111	4,2
**Geometria do VE (massa/altura ^2,7^) (n=2.651)**		
Normal	1344	50,7
Remodelamento concêntrico	815	30,7
Hipertrofia concêntrica	295	11,1
Hipertrofia excêntrica	197	7,4
**Parâmetro Ecocárdio (n=1.419)**		
Normal	486	34,2
Anormal	933	65,8

*AE: átrio esquerdo; VE: ventrículo esquerdo; ASC: área de superfície corporal.*

### Análise de regressão bivariada da associação de alterações ecocardiográficas, clínicas e sociodemográficas com o risco global

Entre as anormalidades ecocardiográficas, DDVE teve associação mais forte com o risco global (≥ 7,5) no primeiro e no segundo períodos do estudo. DDVE também foi a anormalidade mais associada ao risco combinado.

A associação entre HVE e o risco global foi semelhante para ambos os índices de massa (indexado pela ASC e pela altura ^2 , 7^ ). HVE foi associado ao risco global em ambos os períodos, com associação mais forte com o risco global combinado.

O aumento do AE foi a variável com menor associação com o risco global e sem associação com o risco combinado.

Quando os três parâmetros foram analisados em conjunto, a associação com o risco global foi maior no primeiro período do estudo.

Em relação aos demais fatores de risco, não foi observada associação entre atividade física e o risco global. Ao contrário, consumo excessivo de álcool, hipertrigliceridemia, IMC e nível educacional estiveram associados ao risco em ambos períodos do estudo. Associação dessas variáveis com o risco combinado não foi estatisticamente significativa. ( Tabela 3 )

**Tabela 3:** – Associação bivariada entre risco cardiovascular global (em ambos os períodos e risco combinado) e características ecocardiográficas e clínicas no período 1 (2008-2010), n = 2.973

Variável	Risco global (período 1)		Risco global (período 2)		Risco combinado (baixo risco período 1 e alto risco no período 2)	

	RP	IC 95%	RP	IC 95%	RP	IC 95%
**Parâmetro Ecocárdio**						
Anormal	3,26	2,72; 3,91	2,59	2,23; 3,01	2,74	2,00; 3,76
**Função diastólica**						
Com disfunção	2,87	2,49; 3,30	2,55	2,26; 2,89	3,48	2,55; 4,74
**Geometria do VE (massa/ASC)**						
Com hipertrofia	1,54	1,42; 1,67	1,45	1,36; 1,56	2,10	1,59; 2,77
**Geometria do VE (massa/ altura^2,7^)**						
Com hipertrofia	1,48	1,37; 1,60	1,44	1,35; 1,53	1,95	1,56; 2,45
**Volume do AE**						
Aumentado	1,24	1,14; 1,36	1,16	1,07; 1,26	1,16	0,87; 1,55
**Atividade física de lazer**						
Sedentário	1,00	0,94; 1,08	1,02	0,96; 1,08	0,98	0,81; 1,20
**Bebedor excessivo**						
Sim	1,34	1,22; 1,47	1,24	1,14; 1,35	1,04	0,67; 1,61
**Hipertrigliceridemia**						
Sim	1,30	1,22; 1,39	1,20	1,13; 1,27	1,09	0,90; 1,36
**IMC**						
Sobrepeso	1,27	1,17; 1,38	1,19	1,11; 1,28	1,09	0,88; 1,36
Obesidade	1,30	1,19; 1,43	1,22	1,13; 1,33	1,27	0.99; 1.64
**Nível educacional**						
Até o ensino médio completo	1,11	1,04; 1,19	1,08	1,02; 1,15	1,13	0,93; 1,37

*AE: átrio esquerdo; VE: ventrículo esquerdo; ASC: área de superfície corporal; IMC: índice de massa corporal.*

### Análise de regressão logística multivariada da associação entre alterações ecocardiográficas e o risco global

Associação entre as alterações ecocardiográficas e o risco global foi ajustada para algumas variáveis clínicas e sociodemográficas que não faziam parte do desfecho. Como foi observada uma interação de efeito entre DDVE e nível educacional no primeiro modelo de regressão multivariada, DDVE foi estratificada por essa variável. Entretanto, quando a DDVE foi avaliada como variável principal, não foi ajustada para escolaridade.

No primeiro modelo de regressão logística multivariada, observamos que a associação mais forte ocorreu entre o risco global e o parâmetro Ecocardio. A segunda associação mais forte ocorreu entre risco global e DDVE no subgrupo com escolaridade até o ensino médio completo.

Da mesma forma, no segundo modelo de regressão multivariada, a associação mais forte foi observada entre risco global e parâmetro Ecocardio e a segunda mais forte foi observada entre risco global e DDVE (neste modelo, não houve interação com nível educacional).

No terceiro modelo de regressão multivariada, DDVE foi a variável com associação mais forte com o risco global combinado. Neste modelo, associação entre risco global combinado e dilatação do AE não foi significativa. ( Tabela 4 ). O modelo final de regressão é mostrado na tabela S2.

**Tabela 4:** – Regressão logística multivariada * das variáveis ecocardiográficas em relação ao risco global, considerando razões de prevalência (RP) e respectivos intervalos de confiança de 95% (IC95%), n = 2.973

Variáveis (Período 1)	Risco global Período 1 (modelo 1)		Risco global Período 2 (modelo 2)		Risco global combinado Períodos 1 e 2 (modelo 3)	

	RP	IC 95%	RP	IC 95%	RP	IC 95%
**Parâmetro Ecocárdio**						
Anormal	4,01	3,20; 5,03	3,04	2,49; 3,70	2,80	2,02; 3,88
**Disfunção diastólica**						
Presença (todos)	-	-	2,95	2,46; 3,54	3,68	2,63; 5,15
Presença (nível superior **)	2,91	2,31; 3,67	-	-	-	-
Presença (até o ensino médio completo **)	3,88	2,87; 5,26	-	-	-	-
**Hipertrofia do VE**						
Presença	1,72	1,52; 1,94	1,63	1,47; 1,81	2,20	1,62; 3,00
**Dilatação do AE**						
Presença	1,31	1,15; 1,49	1,20	1,07; 1,34	1,16	0,86; 1,57

* ajustado para: hipertrigliceridemia, IMC, atividade física, nível educacional e consumo excessivo de álcool. ** estratificado por nível educacional e ajustado para: hipertrigliceridemia, IMC, atividade física e consumo excessivo de álcool. IMC: índice de massa corporal; AE: átrio esquerdo; VE: ventrículo esquerdo.

Em 2016, após conclusão deste estudo, foram publicadas novas recomendações para avaliação da função diastólica do VE.^17^ Foi possível determinar DDVE aplicando esses critérios em 1.434 indivíduos (48%). A função diastólica foi normal em 829 (57.8%) participantes e entre aqueles que apresentaram disfunção diastólica: 165 (11.5 %) foram classificados como grau I, 18 (1,3%) como grau II e 3 (0,2%) como grau III. Em 419 (29,2%) participantes a função diastólica ou o grau da disfunção diastólica não puderam ser determinadas (dados não apresentados). Os resultados obtidos foram muito semelhantes ao original: DDVE persistiu com associação mais forte com o risco global no primeiro e segundo períodos do estudo (RP = 3,38, IC 95% 2,53- 4,52 e 2,91, IC 95% 2,40- 3,52, respectivamente) assim como com o risco global combinado (RP = 3,24, IC 95% 2,17-4,84).

## Discussão

Observamos uma associação entre alterações ecocardiográficas e alto risco global CV (escore ASCVD ≥ 7,5) nos dois períodos do estudo.

Das três alterações ecocardiográficas analisadas individualmente, DDVE teve a associação mais forte com o risco global nas análises de regressão logística bivariada e multivariada.

Apesar de ser uma coorte de indivíduos assintomáticos e sem DCV prévia, nossos dados revelam que 41,8% dos participantes apresentavam DDVE e, destes, a maioria era grau I ou alteração do relaxamento. Porém, como se trata de uma coorte de idosos (média de idade 60,2 ± 8,8 anos), espera-se maior prevalência de DDVE grau I, pois o envelhecimento normal está associado à diminuição do relaxamento do VE, que pode levar à disfunção diastólica.^18^ Vale ressaltar que Huttin et al.,^19^ mostraram prevalência bem menor de DDVE em indivíduos com idade > 60 anos quando utilizaram as recomendações de 2016 em relação às recomendações anteriores para classificação de DDVE. Da mesma forma, Almeida et al.,^20^ observaram que a prevalência de DDVE em indivíduos com mais de 45 anos foi muito menor quando se utilizou as recomendações de 2016,^17^ do que quando se usou as recomendações de 2009.^13^ Esses autores encontraram prevalência de DDVE de 1,4% e 38,1% quando utilizaram as recomendações de 2016 e 2009, respectivamente. De forma semelhante, no nosso estudo, observamos uma prevalência de DDVE de 13% e 41,8% quando utilizamos as recomendações de 2016 e 2009, respectivamente.

Na análise de regressão logística bivariada da associação entre anormalidades ecocardiográficas e o risco global nos períodos 1 e 2 ou com o risco combinado, todas as três anormalidades foram associadas ao escore ASCVD. Entre as três anormalidades, DDVE mostrou associação mais forte com o risco CV. Tsang et al.,^21^ também concluíram que DDVE foi um preditor de risco mais forte do que dilatação do AE e massa do VE. Da mesma forma, Kardys et al.,^22^ observaram que DDVE foi um preditor mais forte de risco CV do que HVE. Esses autores não encontraram associação entre dilatação do AE e mortalidade por todas as causas.

Quando realizada a regressão logística multivariada, DDVE permaneceu como o parâmetro ecocardiográfico com maior associação com o risco global. Os demais parâmetros ecocardiográficos analisados (HVE e dilatação do AE) mantiveram associação com o risco global nos dois períodos do estudo; entretanto, dilatação do AE não apresentou associação estatisticamente significativa com o risco combinado. No Strong Heart Study,^23^ observou-se que DDVE estava associada à mortalidade CV independentemente das demais alterações ecocardiográficas, semelhante ao resultado do nosso estudo. Da mesma forma, Redfield et al.,^24^ observaram que DDVE estava fortemente associada à mortalidade por todas as causas, demonstrando ser um preditor de risco CV.

O Framingham Heart Study^25^ mostrou que HVE é um preditor de morte por DCV e todas as causas. Recentemente, Desai et al.,^26^ e Lind et al.,^27^ descreveram associação de risco entre HVE e eventos CV, semelhante ao encontrado neste estudo. Ao contrário do estudo atual, no entanto, os estudos anteriores avaliaram desfechos clínicos (doença coronariana, doença cerebrovascular e insuficiência cardíaca) e não desfecho intermediário, como o escore ASCVD.

O aumento do volume do AE foi associado ao risco global (escore ASCVD ≥ 7,5) em ambos os períodos deste estudo, tanto na análise de regressão logística bivariada quanto na multivariada, embora não tenhamos encontrado associação significativa com o risco combinado. Da mesma forma, Laukkanen et al.,^28^ observaram associação entre dilatação do AE e mortalidade; entretanto, quando ajustada para HVE, essa associação não foi significativa. Em outro estudo, Gardin et al.,^29^ observaram associação de dilatação do AE apenas com insuficiência cardíaca, mas não com cardiopatia isquêmica. Já, Bombelli et al.,^30^ concluíram que dilatação do AE é um preditor de eventos CV.

Este estudo demonstrou que anormalidades ecocardiográficas estão associadas a um escore de alto risco (≥ 7,5), enquanto a ausência dessas anormalidades está associada a um escore de baixo risco (<7,5). Assim, esses parâmetros ecocardiográficos podem ser adotados como marcadores de risco, ampliando o leque de achados diagnósticos que permitem estimar precocemente o risco CV dos pacientes. Achados ecocardiográficos são influenciados por alguns fatores de risco que fazem parte do escore ASCVD, principalmente pressão arterial e diabetes, como também podem refletir alterações subclínicas como aterosclerose coronária, hipertrofia miocárdica, dentre outras, que não fazem parte do escore. Optamos por utilizar o escore ASCVD por ser o escore de predição de risco CV mais amplamente utilizado internacionalmente.

### Limitações e perspectivas futuras

Nosso estudo teve algumas limitações. Como a coorte ELSA-Brasil é composta por servidores públicos, a possibilidade de generalizar nossos resultados para a população adulta brasileira é limitada. No entanto, a generalização dos resultados é parcialmente sustentada pelas semelhanças na prevalência de fatores de risco comportamentais e condições crônicas identificadas em dois estudos: ELSA-Brasil^15^ e VIGITEL,^31^ que produziram dados representativos para adultos brasileiros. Outra limitação do estudo é a não utilização da classificação mais atual de DDVE, devido ao fato dos dados terem sido coletados entre 2008 e 2010. No entanto, conforme descrito em Resultados, aplicando as recomendações de 2016 aos nossos dados, observamos essencialmente os mesmos achados, reforçando a importância do parâmetro DDVE para o risco cardiovascular global. Novos estudos de coorte na população brasileira devem ser realizados com o objetivo de identificar se estas alterações ecocardiográficas podem adicionar informação prognóstica incremental ao escore ASCVD.

## Conclusão

Nosso estudo mostrou que anormalidades ecocardiográficas (DDVE, HVE e aumento do volume do AE) estão associadas a um alto risco CV global (escore ASCVD ≥ 7,5) em adultos brasileiros assintomáticos sem DCV prévia. Das três alterações ecocardiográficas, a DDVE mostrou associação mais forte com o risco global. Mais estudos são necessários para avaliar a relação custo-efetividade, a fim de justificar a incorporação dessas variáveis na rotina de estimativa de risco CV e a adoção de medidas de prevenção em nível populacional.

## * Material suplementar

Para informação adicional da tabela S1, por favor, clique aqui .

Para informação adicional da tabela S2, por favor, clique aqui .

## References

[1] World Health Organization (WHO).World health statistics 2018: monitoring health for the SDGs, sustainable development goals. Geneva: 2018. p.1–86

[2] Brasil.Ministério da Saúde.Departamento de Ciência e Tecnologia, Secretaria de Ciência, Tecnologia e Insumos Estratégicos [ELSA Brasil: the greatest epidemiological study in Latin America]. Rev Saude Publica. 2009 Feb;43(1). doi:S0034-89102009000100028 19169569

[3] Goff DC, Lloyd-Jones DM, Bennett G, Coady S, D’Agostino RB, Gibbons R et al . 2013 ACC/AHA Guideline on the Assessment of Cardiovascular Risk. J Am Coll Cardiol. 2014 Jul 1;63(25):2935–59. doi: 10.1016/j.jacc.2013.11.005. Epub 2013 Nov 12. 10.1016/j.jacc.2013.11.005 PMC4700825 24239921

[4] Armstrong AC, Jacobs DR, Gidding SS, Colangelo LA, Gjesdal O, Lewis CE et al. Framingham score and LV mass predict events in young adults: CARDIA study. Int J Cardiol. 2014 Mar 15;172(2):350–5. doi: 10.1016/j.ijcard.2014.01.00 10.1016/j.ijcard.2014.01.003 PMC4068332 24507735

[5] Nayor M, Cooper LL, Enserro DM, Xanthakis V, Larson MG, Benjamin EJ et al. Left ventricular diastolic dysfunction in the community: Impact of diagnostic criteria on the burden, correlates, and prognosis. J Am Heart Assoc. 2018;7(11):e008291. doi: 10.1161/JAHA.117.008291. 10.1161/JAHA.117.008291 PMC6015390 29858363

[6] Aquino EML, Barreto SM, Bensenor IM, Carvalho MS, Chor D, Duncan BB et al. Brazilian Longitudinal Study of Adult Health (ELSA-Brasil): Objectives and Design. Am J Epidemiol. 2012 Feb 15;175(4):315–24. doi: 10.1161/JAHA.117.008291. 10.1093/aje/kwr294 22234482

[7] Cuspidi C, Meani S, Negri F, Giudici V, Valerio C, Sala C et al. Indexation of left ventricular mass to body surface area and height to allometric power of 2.7: Is the difference limited to obese hypertensives? J Hum Hypertens. 2009 Nov;23(11):728–34. doi: 10.1038/jhh.2009.16. 10.1038/jhh.2009.16 19322202

[8] Lang RM, Badano LP, Mor-Avi V, Afilalo J, Armstrong A, Ernande L et al. Recommendations for Cardiac Chamber Quantification by Echocardiography in Adults: An Update from the American Society of Echocardiography and the European Association of Cardiovascular Imaging. Eur Heart J Cardiovasc Imaging. 2015 Mar;16(3):233–71. doi: 10.1093/ehjci/jev014. 10.1093/ehjci/jev014 25712077

[9] Tognon AP, Foppa M, Luft VC, Chambless LE, Lotufo P, El Aouar LMM et al. Reproducibility of Left ventricular Mass by Echocardiogram in the ELSA-Brasil. Arq Bras Cardiol. 2015 Feb; 104(2): 104- 11. doi: 10.5935/abc.20140183. Epub 2014 Nov 28. 10.5935/abc.20140183 PMC4375653 25424165

[10] Devereux RB, Alonso DR, Lutas EM, Gottlieb GJ, Campo E, Sachs I et al. Echocardiographic assessment of left ventricular hypertrophy: Comparison to necropsy findings. Am J Cardiol. 1986 Feb 15;57(6):450–8. doi: 10.1016/0002-9149(86)90771-x. 10.1016/0002-9149(86)90771-x 2936235

[11] Foppa M, Duncan BB, Rohde LEP. Echocardiography-based left ventricular mass estimation. How should we define hypertrophy? Cardiovasc Ultrasound. 2005 Jun 17;3:17. doi: 10.1186/1476-7120-3-17. 10.1186/1476-7120-3-17 PMC1183230 15963236

[12] Marwick TH, Gillebert TC, Aurigemma G, Chirinos J, Derumeaux G, Galderisi M, et al. Recommendations on the Use of Echocardiography in Adult Hypertension: A Report from the European Association of Cardiovascular Imaging (EACVI) and the American Society of Echocardiography (ASE). J Am Soc Echocardiogr. 2015 Jul 1;28(7):727–54. doi: 10.1016/j.echo.2015.05.002. 10.1016/j.echo.2015.05.002 26140936

[13] Nagueh SF, Appleton CP, Gillebert TC, Marino PN, Oh JK, Smiseth OA et al. Recommendations for the evaluation of left ventricular diastolic function by echocardiography. Eur J Echocardiogr.2009;10(2):165-93, doi: 10.1093/ejechocard/jep007. 10.1093/ejechocard/jep007 19270053

[14] Pitanga FJG, Matos SMA, Almeida M da C, Barreto SM, Aquino EML. Leisure-time physical activity, but not commuting physical activity, is associated with cardiovascular risk among ELSA-Brasil participants. Arq Bras Cardiol. 2018;110(1):36–43. doi: 10.5935/abc.20170178. 10.5935/abc.20170178 PMC5831300 29412240

[15] Schmidt MI, Duncan BB, Mill JG, Lotufo PA, Chor D, Barreto SM, et al. Cohort Profile: Longitudinal Study of Adult Health (ELSA-Brasil). Int J Epidemiol. 2015 Feb 1;44(1):68–75. doi: 10.1093/ije/dyu027. Epub 2014 Feb 27. 10.1093/ije/dyu027 PMC4339754 24585730

[16] Ospina R, Amorim LD. Estimation of prevalence ratios using logistic models and confidence intervals with delta and brootstrap methods. 2019.[Internet] [Cited in 2021 May 10] Available from: http://www2.uaem.mx/r-mirror/web/packages/prLogist.

[17] Nagueh SF, Smiseth OA, Appleton CP, Byrd BF, Dokainish H, Edvardsen T et al. Recommendations for the Evaluation of Left Ventricular Diastolic Function by Echocardiography: An Update from the American Society of Echocardiography and the European Association of Cardiovascular Imaging. Eur Heart J Cardiovasc Imaging. 2016 Dec;17(12):1321–60 doi: 10.1093/ehjci/jew082. 10.1093/ehjci/jew082 27422899

[18] Schirmer H, Lunde P, Rasmussen K. Mitral flow derived Doppler indices of left ventricular diastolic function in a general population. The Tromso study. Eur Heart J. 2000;21(16):1376–86. doi: 10.1053/euhj.1999.2036. 10.1053/euhj.1999.2036 10952827

[19] Huttin O, Fraser AG, Coiro S, Bozec E, Selton-Suty C, Lamiral Z et al. Impact of Changes in Consensus Diagnostic Recommendations on the Echocardiographic Prevalence of Diastolic Dysfunction. J Am Coll Cardiol. 2017 Jun 27;69(25):3119–21. doi: 10.1016/j.jacc.2017.04.039. 10.1016/j.jacc.2017.04.039 28641802

[20] Almeida JG, Fontes-Carvalho R, Sampaio F, Ribeiro J, Bettencourt P, Flachskampf FA et al. Impact of the 2016 ASE/EACVI recommendations on the prevalence of diastolic dysfunction in the general population. Eur Heart J Cardiovasc Imaging. 2018;19(4):380–6. doi: 10.1093/ehjci/jex252. 10.1093/ehjci/jex252 29236978

[21] Tsang TSM, Barnes ME, Gersh BJ, Takemoto Y, Rosales AG, Bailey KR et al. Prediction of risk for first age-related cardiovascular events in an elderly population: The incremental value of echocardiography. J Am Coll Cardiol. 2003;42(7):1199–205. doi: 10.1016/s0735-1097(03)00943-4. DOI: 10.1016/j.ijcard.2007.12.031 10.1016/s0735-1097(03)00943-4 14522480

[22] Kardys I, Deckers JW, Stricker BHC, Vletter WB, Hofman A, Witteman JCM. Echocardiographic parameters and all-cause mortality: The Rotterdam Study. Int J Cardiol. 2009;133(2):198–204. doi: 10.1016/j.ijcard.2007.12.031. 10.1016/j.ijcard.2007.12.031 18313776

[23] Bella JN, Palmieri V, Roman MJ, Liu JE, Welty TK, Lee ET et al. Mitral ratio of peak early to late diastolic filling velocity as a predictor of mortality in middle-aged and elderly adults: The strong heart study. Circulation. 2002 Apr 23;105(16):1928–33. doi: 10.1161/01.cir.0000015076.37047.d9. 10.1161/01.cir.0000015076.37047.d9 11997279

[24] Redfield MM, Jacobsen SJ, Burnett JC, Mahoney DW, Bailey KR, Rodeheffer RJ. Burden of systolic and diastolic ventricular dysfunction in the community: Appreciating the scope of the heart failure epidemic. J Am Med Assoc. 2003 Jan 8;289(2):194–202. doi: 10.1001/jama.289.2.194. 10.1001/jama.289.2.194 12517230

[25] Levy D, Garrison RJ, Savage DD, Kannel WB, Castelli WP. Prognostic Implications of Echocardiographically Determined Left Ventricular Mass in the Framingham Heart Study. N Engl J Med 1990;322(22):1561–6. doi: 10.1056/NEJM199005313222203. 10.1056/NEJM199005313222203 2139921

[26] Desai CS, Bartz TM, Gottdiener JS, Lloyd-Jones DM, Gardin JM. Usefulness of Left Ventricular Mass and Geometry for Determining 10-Year Prediction of Cardiovascular Disease in Adults Aged >65 Years (from the Cardiovascular Health Study). Am J Cardiol. 2016;118(5):684–90. doi: 10.1016/j.amjcard.2016.06.016. 10.1016/j.amjcard.2016.06.016 PMC4988901 27457431

[27] Lind L, Sundström J. Change in left ventricular geometry over 10 years in the elderly and risk of incident cardiovascular disease. J Hypertens. 2019;37(2):325–30. doi: 10.1097/HJH.0000000000001897. 10.1097/HJH.0000000000001897 30113528

[28] Laukkanen JA, Kurl S, Eränen J, Huttunen M, Salonen JT. Left atrium size and the risk of cardiovascular death in middle-aged men. Arch Intern Med. 2005;165(15):1788–93. doi: 10.1097/HJH.0000000000001897. 10.1001/archinte.165.15.1788 16087829

[29] Gardin JM, McClelland R, Kitzman D, Lima JAC, Bommer W, Klopfenstein HS et al . M-Mode echocardiographic predictors of six- to seven-year incidence of coronary heart disease, stroke, congestive heart failure, and mortality in an elderly cohort (The Cardiovascular Health Study). Am J Cardiol. 2001;87(9):1051–7. doi: 10.1016/s0002-9149(01)01460-6. 10.1016/s0002-9149(01)01460-6 11348601

[30] Bombelli M, Facchetti R, Cuspidi C, Villa P, Dozio D, Brambilla G et al. Prognostic significance of left atrial enlargement in a general population results of the PAMELA study. Hypertension. 2014;64(6):1205–11. doi: 10.1161/HYPERTENSIONAHA.114.03975. 10.1161/HYPERTENSIONAHA.114.03975 25201892

[31] Brasil.Ministério da Saúde. Departamento de Análise de Situação de Saúde. Vigilância de Fatores de Risco e Proteção para Doenças Crônicas por Inquérito Telefônico, Vigitel - 2010. Brasília;2011.

