# Supplementary material for: Parâmetros Ecocardiográficos Simples são Fortes Preditores de Risco Cardiovascular em Indivíduos Assintomáticos: Coorte Elsa-Brasil
Source: Arq Bras Cardiol. 2022 May 4;118(5):916–24. [Article in Portuguese] doi: 10.36660/abc.20210101 (PMC9368872; doi:10.36660/abc.20210101)
Supplement: Translation

# Translation: Simple Echocardiographic Parameters are Strong Predictors of the Cardiovascular Risk in Asymptomatic Individuals: Elsa-Brasil Cohort

**Authors:** Luciana Pereira Fernandes, Maria da Conceição Chagas de Almeida, Sheila Alvim de Matos, Ana Clara Paixão Campos, Edmundo José Nassri Câmara, Murilo Foppa, Antônio Luiz Pinho Ribeiro, Sandhi Maria Barreto, Roque Aras

**Affiliations:** 1 Brasil Universidade Federal da Bahia, Salvador, BA – Brasil; 2 Brasil Fundação Oswaldo Cruz - Instituto Gonçalo Moniz, Salvador, BA – Brasil; 3 Brasil Universidade Federal do Rio Grande do Sul, Porto Alegre, RS – Brasil; 4 Brasil Universidade Federal de Minas Gerais, Belo Horizonte, MG – Brasil

**Keywords:** Cardiovascular Diseases, Risk Factors, Left Ventricular Diastolic Dysfunction, Left Atrial Volume, Diagnostic, Imaging, Echocardiography/methods, Atherosclerosis, Sedentarism

## Abstract

**Background:**

Several studies have evaluated echocardiographic abnormalities as predictors of cardiovascular risk; however, none have associated the global cardiovascular risk with echocardiographic abnormalities in the Brazilian population.

**Objective:**

This study evaluates the association between the global cardiovascular risk (ASCVD score) and three echocardiographic abnormalities: left ventricular hypertrophy (LVH), left ventricular diastolic dysfunction (LVDD), and increased left atrium (LA) volume.

**Methods:**

The study population was composed of participants from ELSA-Brasil who underwent echocardiography between 2008 and 2010 (n = 2973). They were asymptomatic and had no history of cardiovascular disease. The ASCVD score was calculated in two periods: 2008-2010 and 2012-2014. Prevalence ratios (PR) were estimated with 95% confidence intervals (CI).

**Results:**

There is an association between echocardiographic abnormalities and high global cardiovascular risk (ASCVD score ≥ 7.5) in both study periods, separately. The combined global risk (low risk in the first period and high risk in the second period) was significantly associated only with LVDD (PR = 3.68, CI 95% 2.63–5.15) and LVH (PR = 2.20, 95% CI 1.62–3.00).

**Conclusion:**

Echocardiographic abnormalities (LVDD, LVH, and increased LA volume) are independent predictors of cardiovascular risk in Brazilian adults.

## Introduction

Cardiovascular diseases (CVD) are a global public health problem and a research priority in many countries.^1^ In Brazil, the Longitudinal Study of Adult Health (ELSA-Brasil) aims to investigate the prevalence of chronic non-communicable diseases, especially CVD, and their risk factors in the adult population.^2^ In this context, the identification of CV risk predictors merits investigation.

The most universally used CV risk score is the Atherosclerotic Cardiovascular Disease (ASCVD) score, whose parameters were defined by studies conducted in the United States of America.^3^ Other studies have assessed the predictive ability of echocardiographic abnormalities.^4 , 5^ However, no study has investigated the association of the ASCVD score with echocardiographic abnormalities in the Brazilian population.

Thus, considering the ASCVD score as an intermediate CV outcome, this study assessed the association of echocardiographic abnormalities with ASCVD in asymptomatic individuals without previous CVD involved in two periods of the ELSA-Brasil study: baseline (period 1) and 4 years later (period 2).

## Methods

### Population

The population was composed of ELSA-Brasil participants who underwent echocardiography between 2008 and 2010. These individuals were part of two samples, one random, composed of 10% of the cohort (n = 15,105) and the other, composed of individuals older than 60 years not included in the random sample. At the baseline, those who reported CVD were excluded (left ventricular dysfunction, myocardial infarction, stroke, atrial fibrillation or flutter, and moderate or severe valve disease).

To calculate the ASCVD score, data produced in 2008–2010 and 2012–2014 by ELSA-Brasil were extracted, as described elsewhere.^6^ Echocardiography was performed only in the first period.

As a multicenter study, the research protocol was approved by the ethics committee of each institution and by the National Research Ethics Commission.

### Echocardiography

Echocardiography was performed by trained and certified professionals using a device of the same model (Aplio XG; Toshiba Corporation, Tokyo, Japan) at all six ELSA-Brasil centers, following a standardized technique. Real-time and static images were selected and sent in DICOM (Digital Imaging Communications in Medicine) format to the reading center, where the measurements of the examinations were performed.^6^

We analyzed three echocardiographic parameters: left ventricular hypertrophy (LVH), left ventricular diastolic dysfunction (LVDD) and increased left atrium (LA) volume. LVH was defined according to two criteria: mass index and relative wall thickness (RWT). The mass index was calculated by indexing the LV mass to the body surface area (BSA) or height^2 , 7^ .^7^ The LV mass measurements were done by 2D echocardiography (linear method)^8^ at the reading center,^9^ and the LV mass (in grams) was calculated using the formula 0.80 (1.04 [interventricular septum + LV internal dimension + posterior wall]^3^- [LV internal dimension]^3^) + 0.6, according to Devereux et al.^10^ RWT was calculated using the formula (2 × posterior wall thickness) / (LV inner diameter at the end of diastole).^8^ Using these two criteria, the LV geometry was classified as normal, concentric remodeling, concentric hypertrophy, or eccentric hypertrophy.^11^ The cutoff point for the mass indexed to the BSA was 95 g/m^2^for women and 115 g/m^2^for men.^8^ With the mass indexed to height,^2 , 7^ the cutoff point was 44 g/height^2 , 7^ for women and 48 g/height^2 , 7^ for men.^12^ The cutoff point for the RWT for both sexes was 0.42, considering the two mass index criteria.^8^

Assessment of LV diastolic function was based on the American Society of Echocardiography recommendations published in 2009.^13^ The following measures were used to classify the diastolic function: E/A ratio (ratio of E and A velocities of mitral influx), the velocity of medial and lateral e′ waves (assessed with tissue Doppler), E/e′ ratio, and indexed LA volume. The cutoff points for classifying diastolic dysfunction were as follows: E/A (≤ 0.8, between 0.8 and 2.0, and ≥ 2.0), medial e′ (< 8), lateral e′ (< 10), mean E/e′ (≤ 8,>8 and <13, and ≥ 13), and indexed LA volume (> 34 mL/m^2^). Based on these criteria, diastolic function was classified as normal, diastolic dysfunction grade I or impaired relaxation (normal LA pressure), diastolic dysfunction grade II or pseudonormal (signs of elevated LA pressure), diastolic dysfunction grade III or restrictive filling (significantly elevated LA pressure).

LA volume indexed to the BSA for men and women was categorized as normal (up to 34 mL/m^2^), mildly enlarged (between 35 and 41 mL/m^2^), moderately enlarged (between 42 and 48 mL/m^2^), and severely enlarged (> 48 mL/m^2^).^8^

To jointly analyze the three echocardiographic abnormalities (LVDD, LVH, and increased LA volume), the variable “Echocardio parameter” was created. It was normal when none of the three abnormalities were present and abnormal when at least one of the abnormalities was present.

### Global CV risk score

The global CV risk (ASCVD score) was calculated based on age, sex, race (white, African-American, and others), total cholesterol, high-density lipoprotein (HDL)-cholesterol, systolic blood pressure, treatment for hypertension, presence of diabetes mellitus, and smoking. This score calculates the risk of experiencing a cardiovascular fatal or non-fatal event in 10 years (low < 7.5% and high ≥ 7.5%).^3^ The global risk for each participant in the two study periods was calculated. The combined risk, defined as low risk in the first period and high risk in the second period, was also analyzed.

### Other CV risk factors

In addition to the three echocardiographic abnormalities, we also assessed physical activity, alcohol consumption, serum triglyceride level, body mass index (BMI), and educational level. Concerning physical activity, participants were categorized as sedentary/not very active (< 150 min/week of moderate physical activity) or physically active/very active (at least 150 min/week of moderate physical activity).^14^ Concerning alcohol consumption, the categories were excessive or non-excessive drinking (> 210 or < 210 g of alcohol per week for men and > 140 or < 140 g of alcohol per week for women). Concerning serum triglyceride level, the categories were < 150 or ≥ 150 mg/dL. With respect to BMI, the participants were classified as obese (≥ 30 kg/m^2^), overweight (≥ 25and < 30 kg/m^2^), or eutrophic (< 25 kg/m^2^).^15^ Finally, two categories of educational level were considered: up to complete high school and university degree.

### Statistical analysis

Initially, a descriptive analysis of the participants’ sociodemographic, clinical, and echocardiographic profiles was performed, considering absolute and relative frequencies. Subsequently, a bivariate logistic regression analysis was performed to verify the association between the echocardiographic, clinical, and sociodemographic characteristics and the global CV risk in each study period and the combined risk. Prevalence ratios (PRs) were estimated with 95% confidence intervals (CIs), using the CS command of the STATA version 12 software. The prLogistic package of R version 3.5.1 software was used for the multivariate logistic regression analysis to estimate PRs using logistic models and CIs using the delta and bootstrap methods.^16^ Effect modification was evaluated for the following covariates: education, physical activity, excessive alcohol consumption, triglyceride level, and BMI. The likelihood ratio test was used in the multivariate logistic regression model, incorporating product terms (interaction) between the main association and each covariate. A p-value of < 5% in the likelihood ratio test was indicative of an effect change.

## Results

### Sociodemographic and clinical characteristics of the study population at baseline

After excluding individuals who reported having a CV disease, the final study sample comprised 2973 participants, with an average age of 60.26 ± 8.89 years, mainly white and black (56.4% and 39.9%), and most with a university degree (56.7%). Sociodemographic and clinical characteristics of the participants at the baseline of the study are shown in table 1 . Clinical characteristics of the participants in period 2 of the study are shown in table S1.

**Table 1:** – Clinical and sociodemographic characteristics of the participants, n=2973, at the baseline of the study

Clinical and sociodemographic characteristics	n	%
**Sex**		
Men	1358	45.7
Women	1615	54.3
**Ages (years)**		
35- 44	220	7.4
45- 54	487	16.4
55- 64	1240	41.7
65- 74	1025	34.5
**Race**		
White	1658	56.4
Black	1174	39.9
Others	109	3.7
**Educational level**		
University degree	1686	56.7
Up to complete high school	1287	43.3
**Hypertension**		
Yes	1440	48.5
**BMI**		
Overweight	1262	42.4
Obesity	659	22.2
**Fasting blood glucose**		
(≥126 mg/dl)	367	12.3
**Glycated hemoglobin**		
(≥6.5)	323	10.9
**Total cholesterol**		
(> 200 mg/dl **)**	1847	62.2
**Low HDL**		
Yes	509	17.1
**High triglycerides**		
Yes	940	31.6
**Excessive drinker**		
Yes	223	7.5
**Smoking**		
Ex-smoker	1047	35.2
Smoker	296	10.0
**Physical activity**		
Sedentary	1262	42.8

*BMI: body mass index; HDL: high-density lipoprotein.*

### Global CV risk (ASCVD score)

The ASCVD score was assessed as an intermediate clinical outcome in the two study periods. Association of global risk with separate and grouped echocardiographic parameters was analyzed using sociodemographic (educational level) and clinical (physical activity, alcohol intake, hypertriglyceridemia, and BMI) factors. As age, sex, race/color, total cholesterol, HDL-cholesterol, hypertension, diabetes mellitus, and smoking are part of the construction of this risk score, the association with these variables was not evaluated.

The global risk was < 7.5% (low) in 1398 participants (47%) in the first period and 1034 participants (38.3%) in the second period, and ≥ 7.5% (high) in 1575 participants (53%) in the first period and 1665 participants (61.7%) in the second period. The combined risk (low risk in the first period and high risk in the second period) was present in 312 participants (23.7%).

### Echocardiographic characteristics

In 50.8% of the participants’ diastolic function was considered normal, and in 41.8% as abnormal (of these, 31.2% were grade I). In 7.4% of the participants, the diastolic function or the degree of diastolic dysfunction could not be determined.

The LA volume was increased in 15.6% of 2438 participants.

LVH was classified based on two types of mass indexing: BSA (in 2670 participants) and height^2^ (in 2651 participants). The proportion of participants with LVH was higher when indexing by height was used (18.5% versus 10.6%), mainly at the expense of concentric hypertrophy (11.1% versus 6.4%).

In the simultaneous analysis of the three parameters, 65.8% of the participants presented at least one and 34.2% had none of the three abnormalities. ( Table 2 )

**Table 2:** – Echocardiographic characteristics of the participants in period 1, n = 2973

Characteristic	n	%
**Diastolic function (n=1384)**		
No dysfunction	703	50.8
Type I dysfunction	432	31.2
Type II dysfunction	147	10.6
Indeterminate	102	7.4
**LA volume (n=2438)**		
Normal	2058	84.4
Mildly enlarged	281	11.5
Moderately enlarged	73	3.0
Severely enlarged	26	1.1
**LV geometry (mass/BSA) (n=2670)**		
Normal	1449	54.3
Concentric remodeling	940	35.2
Concentric hypertrophy	170	6.4
Eccentric hypertrophy	111	4.2
**LV geometry (mass/height^2.7^) (n=2651)**		
Normal	1344	50.7
Concentric remodeling	815	30.7
Concentric hypertrophy	295	11.1
Eccentric hypertrophy	197	7.4
**Echocardio Parameter (n=1419)**		
Normal	486	34.2
Abnormal	933	65.8

*LA: left atrium; LV: left ventricle; BSA: body surface area.*

### Bivariate regression analysis of the association of echocardiographic, clinical, and sociodemographic abnormalities with global risk

Among the echocardiographic abnormalities, LVDD had the strongest association with global risk (≥7.5) in the first and second) study periods. LVDD was also the abnormality that was most associated with the combined risk.

The association between LVH and global risk was similar for both mass indices (indexed to BSA and indexed to height^2 , 7^ ). LVH was associated with global risk in both periods, with the strongest association being with the combined global risk.

Increased LA volume was the variable with the lowest association with global risk and without association with the combined risk.

When the three parameters were analyzed together (variable Echocardio parameter), the association with global risk was greater in the first study period.

No association was observed between physical activity and global risk concerning the other risk factors. On the contrary, excessive drinking, high triglycerides, BMI, and educational level (university degree as a reference) were associated with risk in both study periods. The Association of these variables with the combined risk was not statistically significant. ( Table 3 )

**Table 3:** – Bivariate association between global cardiovascular risk (in both periods and combined risk) and echocardiographic and clinical characteristics in period 1 (2008 - 2010), n = 2973

Variable	Global risk (period 1)		Global risk (period 2)		Combined risk (low-risk period 1 and high-risk period 2)	

	PR	CI 95%	PR	CI 95%	PR	CI 95%
**Echocardio Parameter**						
Abnormal	3.26	2.72; 3.91	2.59	2.23; 3.01	2.74	2.00; 3.76
**Diastolic function**						
With dysfunction	2.87	2.49; 3.30	2.55	2.26; 2.89	3.48	2.55; 4.74
**LV geometry (mass/BSA)**						
With hypertrophy	1.54	1.42; 1.67	1.45	1.36; 1.56	2.10	1.59; 2.77
**LV geometry (mass/ height^2.7^)**						
With hypertrophy	1.48	1.37; 1.60	1.44	1.35; 1.53	1.95	1.56; 2.45
**LA volume**						
Increased	1.24	1.14; 1.36	1.16	1.07; 1.26	1.16	0.87; 1.55
**Leisure-time physical activity**						
Sedentary	1.00	0.94; 1.08	1.02	0.96; 1.08	0.98	0.81; 1.20
**Excessive drinker**						
Yes	1.34	1.22; 1.47	1.24	1.14; 1.35	1.04	0.67; 1.61
**High triglycerides**						
Yes	1.30	1.22; 1.39	1.20	1.13; 1.27	1.09	0.90; 1.36
**BMI**						
Overweight	1.27	1.17; 1.38	1.19	1.11; 1.28	1.09	0.88; 1.36
Obesity	1.30	1.19; 1.43	1.22	1.13; 1.33	1.27	0.99; 1.64
**Educational level**						
Up to complete high school	1.11	1.04; 1.19	1.08	1.02; 1.15	1.13	0.93; 1.37

*LA: left atrium; LV: left ventricle; BSA: body surface area; BMI: body mass index.*

### Multivariate logistic regression analysis of the association between echocardiographic abnormalities and global risk

The association between echocardiographic abnormalities and global risk was adjusted for some clinical and sociodemographic variables that were not part of the outcome (global risk). The dysfunction was stratified using this variable because of the effect interaction between LVDD and educational level in the first multivariate regression model using the risk in the first period. However, when LVDD was assessed as the main variable, it was not adjusted for education.

In the first multivariate logistic regression model (echocardiographic variables and global risk in the first period), we observed that the strongest association occurred between global risk and the Echocardio parameter. The second strongest association occurred between global risk and LVDD in the participants with an education level of up to complete high school.

Likewise, the strongest association was observed in the second multivariate logistic regression model between global risk and the Echocardio parameter. The second strongest association was observed between global risk and LVDD (in this model, there was no interaction with educational level).

In the third multivariate logistic regression model (echocardiographic variables and combined global risk), LVDD was the variable with the strongest association with combined global risk. In this model, the association between combined global risk and LA dilation was not significant. ( Table 4 ). The final regression model is shown in table S2.

**Table 4:** – Multivariate logistic regression* of echocardiographic variables in relation to the global risk, considering prevalence ratios (PR) and respective 95% confidence intervals (95% CI), n = 2973

Variables	Global risk Period 1 (model 1)		Global risk Period 2 (model 2)		Combined global risk Periods 1 and 2 (model 3)	

	PR	CI 95%	PR	CI 95%	PR	CI 95%
**Echocardio parameter**						
Abnormal	4.01	3.20; 5.03	3.04	2.49; 3.70	2.80	2.02; 3.88
**Diastolic dysfunction**						
Presence (all)	-	-	2.95	2.46; 3.54	3.68	2.63; 5.15
Presence (University degree **)	2.91	2.31; 3.67	-	-	-	-
Presence (Up to complete high school **)	3.88	2.87; 5.26	-	-	-	-
**LV hypertrophy**						
Presence	1.72	1.52; 1.94	1.63	1.47; 1.81	2.20	1.62; 3.00
**LA dilation**						
Presence	1.31	1.15; 1.49	1.20	1.07; 1.34	1.16	0.86; 1.57

*adjusted for: high triglycerides, BMI, physical activity, educational level and excessive drinker. **stratified by educational level and adjusted for: high triglycerides, BMI, physical activity and excessive drinker. LA: left atrium; LV: left ventricle.

In 2016, after completing this study, the new recommendations for the evaluation of LV diastolic function were published.^17^ It was possible to determine LVDD applying these criteria in 1434 individuals (48%). Diastolic function was normal in 829 (57.8%) participants, and among those who had diastolic dysfunction: 165 (11.5%) were classified as type I, 18 (1.3%) as type II and 3 (0.2%) as type III. In 419 (29.2%) participants, the diastolic function or the degree of diastolic dysfunction could not be determined (data not shown). The obtained results were very similar to the original: LVDD persisted with the strongest association with global risk in the first and second study periods (PR= 3.38, 95% CI 2.53; 4.52 and 2.91, 95% CI 2.40; 3.52, respectively) as well as with the combined global risk (PR = 3.24, 95 % CI 2.17; 4.84).

## Discussion

We observed an association between echocardiographic abnormalities and high global CV risk (ASCVD score ≥ 7.5) in the two study periods.

Of the three echocardiographic abnormalities analyzed individually, LVDD had the strongest association with global risk in the bivariate and multivariate logistic regression analyses.

Despite being a cohort of asymptomatic individuals without previous CVD, our data reveal that 41.8% of the participants had LVDD. Of these, the majority were grade I or impaired relaxation. However, in the case of a cohort of older people (mean age 60.2 ± 8.8 years), a higher prevalence of LVDD grade I was expected because normal aging is associated with a decrease in LV relaxation, leading to diastolic dysfunction.^18^ It is worth mentioning that Huttin et al.^19^ showed a much lower prevalence of LVDD in individuals aged > 60 years when they used the 2016 recommendations concerning previous recommendations for classifying LVDD. Likewise, Almeida et al.^20^ observed that the prevalence of LVDD in individuals older than 45 years was much lower when using the 2016 recommendations^17^ than when using the 2009 recommendations.^13^ These authors found a prevalence of LVDD of 1.4% and 38.1% when they used the 2016 and 2009 recommendations, respectively. Similarly, in our study, we observed a prevalence of LVDD of 13% and 41.8% when using the recommendations of 2016 and 2009, respectively.

In the bivariate logistic regression analysis of the association between echocardiographic abnormalities and the global risk in periods 1 and 2or the combined risk, all three abnormalities were associated with the ASCVD score. LVDD showed the strongest association with the CV risk among the three abnormalities. Tsang et al.^21^ also concluded that LVDD was a stronger risk predictor than LA dilation and LV mass. Likewise, Kardys et al.^22^ observed that LVDD was a stronger predictor of CV risk than LVH. These authors found no association between LA dilation and mortality from all causes.

When multivariate logistic regression was performed, LVDD remained the echocardiographic parameter with the greatest association with global risk. The other echocardiographic parameters analyzed (LVH and LA dilation) maintained associations with the global risk in both study periods; however, LA dilation did not present a statistically significant association with the combined risk. In the Strong Heart Study,^23^ it was observed that LVDD was associated with CV mortality regardless of the other echocardiographic abnormalities, similar to the result of our study. Likewise, Redfield et al.^24^ observed that LVDD was strongly associated with mortality from all causes, thus proving to be a predictor of CV risk.

The Framingham Heart Study^25^ showed that LVH is a predictor of death from CVD and all causes. Recently, Desai et al.^26^ and Lind et al.^27^ described a risk association between LVH and CV events, similar to what was found in this study. Unlike the current study, however, those previous studies evaluated clinical outcomes (coronary heart disease, cerebrovascular disease, and heart failure) and not an intermediate outcome such as the ASCVD score.

Increased LA volume was associated with global risk (ASCVD score ≥ 7.5) in both periods of the current study, both in the bivariate and multivariate logistic regression analyses. However, we did not find a significant association with the combined risk. Similarly, Laukkanen et al.^28^ observed an association between LA dilation and mortality; however, when adjusted for LVH, this association was not significant. In another study, Gardin et al.^29^ observed an association of LA dilation only with heart failure but not with ischemic heart disease. Bombelli et al.^30^ concluded that LA dilation is a predictor of CV events.

This study demonstrated that echocardiographic abnormalities are associated with a high-risk score (≥ 7.5), whereas the absence of these abnormalities is associated with a low-risk score (< 7.5). Thus, these echocardiographic parameters can be adopted as risk markers, expanding the range of diagnostic findings that allow the early estimation of CV risk in patients. Echocardiographic findings are influenced by some risk factors part of the ASCVD score, mainly blood pressure and diabetes. They may also reflect subclinical changes such as coronary atherosclerosis and myocardial hypertrophy, among others, that are not part of the score. We chose to use the ASCVD score in our study because it is the 10-year CV risk prediction score most widely used internationally.

### Study limitations and future perspectives

Our study had some limitations. As the ELSA-Brasil cohort comprises civil servants, the possibility of generalizing our results to the Brazilian adult population is limited. However, the generalization of the results is partly supported by the similarities in the prevalence of behavioral risk factors and chronic conditions identified in two studies: ELSA-Brasil^15^ and VIGITEL,^31^ which produced representative data for Brazilian adults. Another limitation of the study is the failure to use the most current classification of LVDD because the data were collected between 2008 and 2010. However, as we described in Results, applying the 2016 recommendations to our data, we observed essentially the same findings, reinforcing the importance of the LVDD parameter for the global cardiovascular risk. New cohort studies in the Brazilian population should be carried out to identify whether these echocardiographic abnormalities can add incremental prognostic information to ASCVD.

## Conclusion

Our study showed that echocardiographic abnormalities (LVDD, LVH, and increased LA volume) are associated with a high global CV risk (ASCVD score ≥ 7.5) in asymptomatic Brazilian adults without previous CVD. Of the three echocardiographic abnormalities, LVDD showed the strongest association with the global risk. More studies are needed to assess the cost-effectiveness ratio to justify the incorporation of these variables in the CV risk estimation routine and the adoption of prevention measures at the population level.

## * Supplemental Materials

For additional information of table S1, please click here .

For additional information of table S2, please click here .
